# The impact of African ethnicity and migration on pregnancy in women living with HIV in the UK: design and methods

**DOI:** 10.1186/1471-2458-12-596

**Published:** 2012-08-02

**Authors:** Shema Tariq, Alex Pillen, Pat A Tookey, Alison E Brown, Jonathan Elford

**Affiliations:** 1 School of Health Sciences, City University London, 20 Bartholomew Close, London, EC1A 7QN, United Kingdom; 2 Department of Anthropology, University College London, 14 Taviton Street, London, WC1H 0BW, United Kingdom; 3 MRC Centre of Epidemiology for Child Health, UCL Institute of Child Health, 30 Guilford St, London, WC1N 1EH, United Kingdom; 4 HIV & STI Department, Health Protection Services - Colindale, Health Protection Agency, 61 Colindale Avenue, London, NW9 5EQ, United Kingdom

**Keywords:** HIV, Pregnancy, Migrants, Ethnicity, Mixed methods research

## Abstract

**Background:**

The number of reported pregnancies in women with diagnosed HIV in the UK increased from 80 in 1990 to over 1400 in 2010; the majority were among women born in sub-Saharan Africa. There is a paucity of research on how social adversity impacts upon pregnancy in HIV positive women in the UK; furthermore, little is known about important outcomes such as treatment uptake and return for follow-up after pregnancy. The aim of this study was to examine pregnancy in African women living with HIV in the UK.

**Methods and design:**

This was a two phase mixed methods study. The first phase involved analysis of data on approximately 12,000 pregnancies occurring between 2000 and 2010 reported to the UK’s National Study of HIV in Pregnancy and Childhood (NSHPC). The second phase was based in London and comprised: (i) semi-structured interviews with 23 pregnant African women living with HIV, 4 health care professionals and 2 voluntary sector workers; (ii) approximately 90 hours of ethnographic fieldwork in an HIV charity; and (iii) approximately 40 hours of ethnographic fieldwork in a Pentecostal church.

**Discussion:**

We have developed an innovative methodology utilising epidemiological and anthropological methods to explore pregnancy in African women living with HIV in the UK. The data collected in this mixed methods study are currently being analysed and will facilitate the development of appropriate services for this group.

## Background

### HIV infection in the UK

In 2010, an estimated 91,500 people were living with HIV in the United Kingdom (UK) [1]; the number continues to grow, mainly due to increased life expectancy as a result of antiretroviral therapy (ART) *ibid*. In the UK, HIV prevalence is elevated both among men who have sex with men (MSM) and African-born heterosexual men and women [1]; for both groups, 5% are estimated to be living with HIV *ibid*. Within the UK itself there is substantial geographical variation in diagnosed HIV prevalence: 54 local authorities have a diagnosed HIV prevalence of greater than 2 per 1000 population aged 15–59 years; 29 of these local authorities are in London [1].

### HIV in African communities in the UK

There were an estimated 29,200 people born in Africa living with HIV in the UK by the end of 2010, 66% (n = 19,300) of whom were women [1]. The majority of Africans newly diagnosed with HIV in the UK originate from East Africa, although the epidemic has become more diverse over time. The proportion of Africans diagnosed with HIV who are from East Africa has fallen from just under 75% in 2001 to approximately 50% in 2010, whilst in the same time period there was a significant increase in diagnoses in West Africans to a point in 2010 when almost 1 in 3 Africans diagnosed with HIV in the UK were West African (Meaghan Kall, Health Protection Agency, personal communication, 25 June 2012).

Studies have shown that African patients are more likely to present to medical services at a later stage of HIV infection, with advanced disease and greater immune suppression [1,2]. This is due to a number of factors including lack of perceived risk, fear of stigma and discrimination, lack of HIV testing in general medical settings, and anxieties regarding medical bills for HIV care [3,4]. African heterosexual patients are also more likely to be lost to follow-up from medical care than white MSM [5,6].

Many Africans living with HIV in the UK have a high level of social need [7,8] including financial difficulty [7], social isolation [9] and insecure immigration status [10,11]. These are likely to impact on patients’ access to healthcare.

### HIV and pregnancy in the UK

There has been a substantial increase in the number of HIV-infected women reported as pregnant to the NSHPC, the UK and Ireland’s national surveillance programme for HIV in pregnancy and childhood: a 17-fold increase from 82 in 1990 to over 1400 a year since 2006 [12] , with approximately 80% of pregnancies reported in recent years in women born in Sub-Saharan Africa [13].

The combination of a routine offer of antenatal HIV screening to all pregnant women, use of ART for the prevention of mother-to-child transmission (MTCT), planned mode of delivery and advice to avoid breastfeeding has resulted in a decline in national MTCT rates from approximately 20% in diagnosed women in 1990 [14] to 1.0% in 2000–2006 [15]. The rate is even lower (0.8%) in women who have received at least 14 days of antiretroviral therapy prior to delivery [15].

In the UK, there is a paucity of data on antenatal and postnatal outcomes other than mother-to-child transmission and gestational age at delivery. Rates of virological suppression in pregnancy have been estimated at between 67 to 75% [16-18]. Looking at access to health services, a small study in London demonstrated that up to 65% of mothers living with HIV failed to return for HIV care after delivery [19]. In terms of care prior to delivery, no studies in the UK examining antenatal care access in HIV-infected women have been identified. Furthermore, there is little work specifically focusing on pregnancy in African women living with HIV in the UK, despite this being the largest group.

Qualitative studies have an important role in elucidating reasons for disparity in outcomes and access, and have provided insights into the experiences of pregnant women living with HIV. However, the vast body of qualitative work on pregnancy and HIV has been conducted in North America and Sub-Saharan Africa and may not be applicable in the UK. Few qualitative studies have explored the experience of pregnancy in women living with HIV in the UK, but those which have [20-22] demonstrated high levels of social isolation and stigma in pregnant women living with HIV. These studies highlighted women’s pervasive fear of transmitting HIV to their child and their acceptance of interventions to prevent this, but also revealed the difficulties that accompany these interventions. Two of these studies included African participants, although sample sizes were small and they remain unpublished [20,21]. Wilson’s Glasgow-based study [22] was larger but the participants were exclusively white British, presenting difficulties in extrapolating results to an ethnically diverse clinic population.

### Rationale for this study

Few studies have explored the impact of African ethnicity and migration on pregnancy in women living with HIV [15,20,21,23-26]. This is a complex area of study requiring a range of investigatory approaches. We believe that there is an urgent need for large-scale work, both quantitative and qualitative, exploring the multi-faceted relationship between HIV and pregnancy among African migrant women in the UK.

### Study objectives

This study aimed to examine disparities in clinical outcomes and access to services among pregnant African women living with HIV in the UK, and to explore how their experiences of pregnancy may contribute to any identified disparities. For the purposes of this study African was defined as being of black ethnicity and having been born in sub-Saharan Africa. Women of mixed, white or Asian ethnicities who were born in sun-Saharan Africa were not defined as African.

The primary objectives were to:

explore the association of: (i) ethnicity, (ii) African region of birth, and (iii) duration of residence in the UK with:

Time of antenatal booking in women living with HIV

Maternal uptake of antiretroviral therapy

Detectable maternal HIV viral load at delivery

Mother-to-child transmission of HIV

Return for HIV care in the calendar year following pregnancy

investigate possible contextual factors that may contribute to any identified disparities in the outcomes above, using qualitative data

describe the experience of HIV and pregnancy in individual women’s lives

## Methods and design

### Overall study design

A mixed methods research approach was designed to meet these objectives. The most widely accepted definition of mixed methods research is the “collecting, analysing, and mixing (of) both quantitative and qualitative data in a single study or a series of studies” [27]. The underlying assumption of mixed methods research is that it can address a research question more comprehensively than using either quantitative or qualitative methods alone. Within the field of HIV, a number of recent studies have illustrated the role of mixed methods research in engaging with the complex nature of HIV care [28,29].

#### Mixed methods model

**Figure 1:**
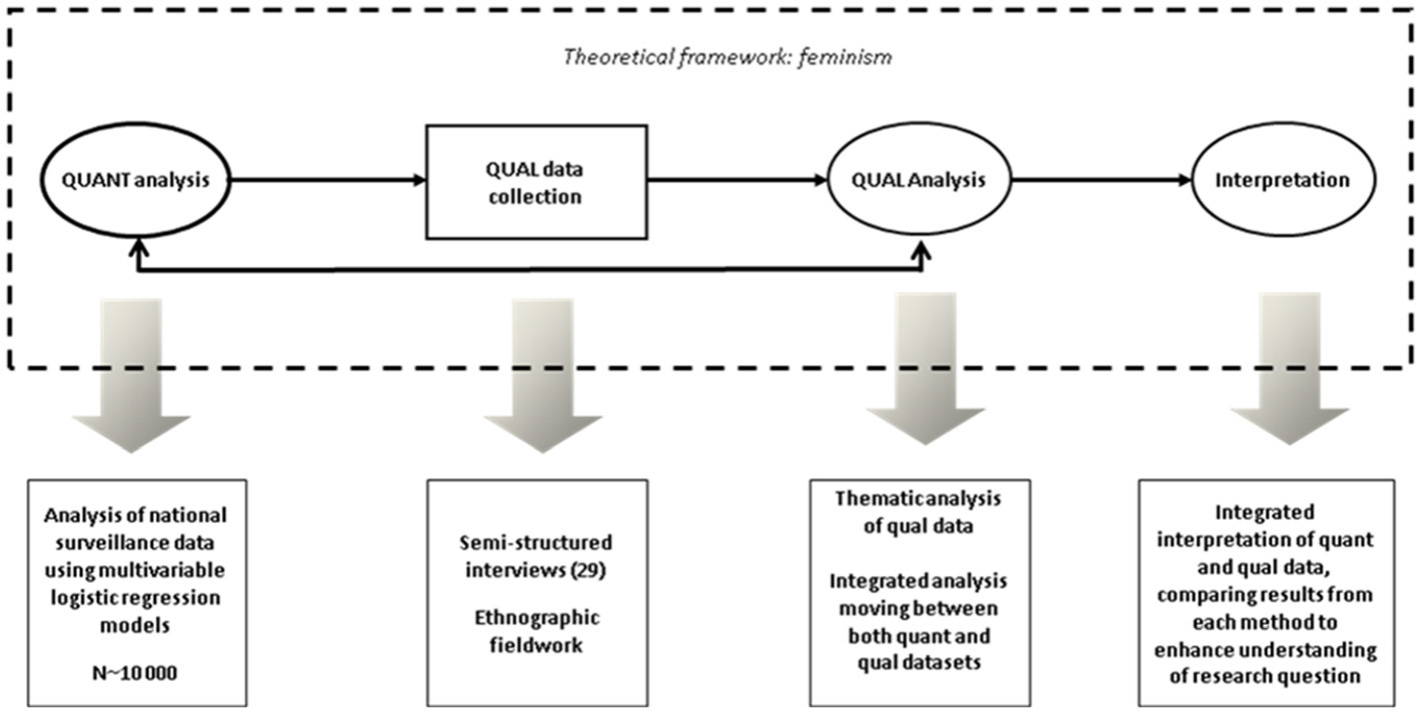
Mixed methods design for the study.

The present study combines epidemiological and anthropological methods, with each approach given equal weight. We used a sequential explanatory model [27] (Figure1). The first phase was quantitative, comprising analysis of linked national surveillance data. This was followed by a qualitative phase which sought to explain and contextualise the findings from the first phase whilst highlighting other important aspects of women’s experience. This qualitative phase comprised semi-structured interviews and participant observation. The study model was embedded within a framework of feminism. This theoretical lens informed the methods, analysis and interpretation throughout the study. Feminist research has a commitment to non-essentialism, which is an understanding that gender, and other social and cultural groups, are not homogeneous or concrete. By exploring differences among women, and among African women, we have attempted to move away from universal gender and ethnicity categories that dominate most epidemiological literature. Furthermore, we have used qualitative methods in an effort to engage with and document women’s experiences, whilst recognising the importance of quantitative research in producing generalisable findings that may inform practice.

#### Rationale for a mixed methods approach

We chose a mixed methods approach as we were studying a complex biosocial phenomenon and felt that a combination of a variety of methods would enhance our understanding. Furthermore, the quantitative findings would inform our sampling and methods in the qualitative phase and the qualitative data would contextualise the quantitative results. We also felt that this approach would place the voices of women living with HIV at the centre of this study.

### Quantitative phase

The quantitative phase comprised secondary analysis of epidemiological data from the National Study of HIV in Pregnancy and Childhood (NSHPC). The analysis of postnatal attendance for HIV care included data from the Survey of Prevalent Infections Diagnosed (SOPHID).

#### The national study of HIV in pregnancy and childhood (NSHPC)

The NSHPC, coordinated at the University College London (UCL) Institute of Child Health (ICH), is a population-based active surveillance study that aims to include all HIV infected women seeking antenatal care in the UK and Ireland [12]. By the end of 2011 data on approximately 15 000 pregnancies since 1990 were available. Pregnancies in HIV-infected women diagnosed by the time of delivery, and infants born to infected women, are reported through two active parallel schemes managed in collaboration with the Royal College of Obstetricians and Gynaecologists and the British Paediatric Surveillance Unit [30]; full methods are described elsewhere [13]. Data collected include: maternal demographics, maternal laboratory results, clinical management of pregnancy and delivery, pregnancy outcome, and infant HIV status.

Pregnancies reported to the NSHPC were included in this study if year of delivery or estimated date of delivery (EDD) was between 1990 and 2010. Reports from Ireland were excluded, as this study focused on the UK. Reports were excluded if: the report concerned a twin or triplet who was not the first-born (to avoid duplication of information on the mother), the child was born outside the UK, or if there were no data on maternal ethnicity or country of birth (the key variables of interest).

For analyses of primary outcomes, pregnancies were included if year of delivery or EDD was 2000 or after, corresponding with wider use of ART in pregnancy and more consistency in clinical practice and monitoring than in the previous decade. Pregnancies were also excluded from these analyses if the mother was diagnosed with HIV after delivery. There were further exclusion criteria specific to each analysis and therefore numbers varied depending on the outcome examined.

#### The survey of prevalent HIV infections diagnosed (SOPHID)

SOPHID is an annual cross-sectional survey of all individuals aged 15 and above with diagnosed HIV infection who attend for National Health Service (NHS) HIV care in the UK within a calendar year [31]. It is coordinated by the Heath Protection Agency and was introduced in 1995. Data collected include: site of care, infection route, ethnicity and date last seen (or date of death) as well as clinical markers*.*

#### Record linkage

We created a combined dataset using NSHPC and SOPHID data to explore whether a woman returned for HIV care anywhere in England, Wales and Northern Ireland in the year following pregnancy. Women known by the NSHPC to be pregnant between 1999 and 2009 were matched to the SOPHID dataset by year of pregnancy. A hierarchical matching strategy was implemented using limited identifiers collected in both systems such as: sex; date of birth; residential information; strategic health authority; country of birth; and date of HIV diagnosis. Potential duplicate reports were identified and not included in the analysis. We excluded pregnancies in women reported from Scotland to the NSHPC or reports to SOPHID from Scotland as prior to 2008 Scottish reports to SOPHID were not linked over time, and it was therefore difficult to establish links between records in the same patient prior to 2008. Pregnancies in women known to have moved abroad during their pregnancy were also excluded. There were 9834 eligible NSHPC pregnancies between 1999 and 2009. In 8695 (88.4%) pregnancies we were able to match the mother to a record in SOPHID.

### Qualitative phase

The qualitative phase of the study comprised semi-structured interviews and ethnographic fieldwork.

#### Semi-structured interviews

The first author conducted semi-structured interviews with pregnant African women living with HIV, healthcare providers, and staff from voluntary sector organisations.

**Figure 2:**
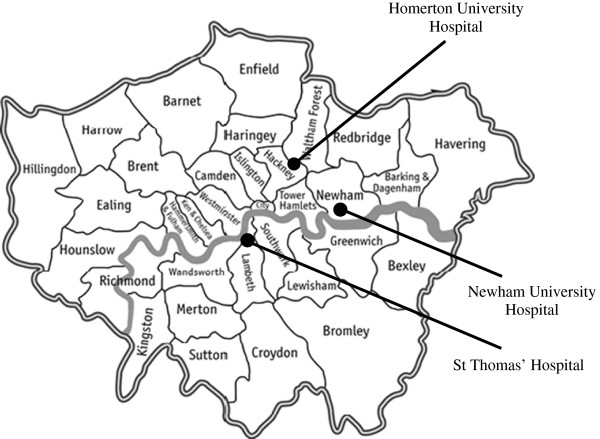
Map of London with NHS study sites.

Twenty-three pregnant women were recruited from three specialist NHS HIV antenatal clinics in London between October 2010 and October 2011 (Figure2). These three sites are among the five hospitals reporting the largest numbers of pregnancies in HIV infected women between 2000 and 2010 (data extracted from most recent NSHPC dataset). Each centre looks after approximately 40 to 50 pregnant women living with HIV each year. They are in boroughs of great ethnic diversity, and also substantial deprivation with all three classified as among the twenty most deprived local authorities in England [32].

**Figure 3:**
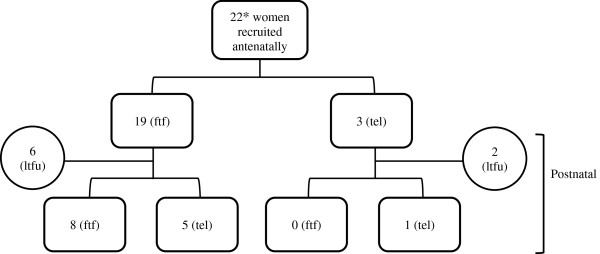
Semi-structured interview recruitment.

Healthcare professionals working at these sites identified and approached women attending for HIV antenatal care who were eligible for the study. Women were eligible if they were of black African ethnicity, were born in sub-Saharan Africa, were diagnosed with HIV and were pregnant (at any gestation). The first author was based on site during HIV antenatal clinics and was able to discuss the study further with women who were interested, providing them with an information sheet. If a woman wished to participate we found a convenient time for her to attend to be interviewed. Written informed consent was obtained prior to each interview. Face-to-face interviews (n = 20) were conducted in a private room in the hospital site with an interpreter present if required (n = 1). Topics covered included experience of pregnancy; attitudes to medical interventions; psychosocial support; experience of healthcare during pregnancy; and stigma and discrimination. A minority of initial interviews (n = 3) were conducted by telephone due to participant preference (Figure3). Telephone interviewing is increasingly used in health research [33] and is considered effective and especially useful in ‘hard to reach’ populations such as mothers with young children.

A follow-up interview after birth was arranged with each woman who had been interviewed during pregnancy (n = 22). Serial interviews can result in the development of increased trust between researcher and participant, facilitating more open discussion [34]. Furthermore, given that the transition between pregnancy and motherhood is a dynamic time, we felt that serial interviews might better capture this changing experience. The follow-up interview occurred at a time convenient for the participant, up to one year after delivery. Topics included experience of delivery; experience of infant feeding; support at home after delivery; and engagement with HIV services after delivery. Some of these interviews (n = 6) were conducted by telephone due to women’s difficulty in attending for interview when caring for a newborn infant.

In total 23 women were recruited for the qualitative phase of the study over one year, the majority of them (22) recruited whilst pregnant. One participant had been approached whilst pregnant but chose to defer her interview until after delivery due to poor health. This sample size is typical of much qualitative research and allowed us to reach data saturation. Of the 22 women recruited during pregnancy, 14 (64%) were interviewed postnatally. We were unable to contact the remaining 8 women or they declined to be interviewed again.

Interviews were recorded on a digital voice recorder where possible, unless a participant had objections to this. In these rare cases, extensive contemporaneous written notes were taken.

Initial sampling was purposive as we attempted to recruit women from a range of African regions, with a range of migration histories, and at different stages of diagnosis (Table1). Sampling was also guided by the initial quantitative results in order to explore emerging findings. As the study progressed the sampling became theoretical as we selected potential cases to test emergent themes and theories.

**Table 1:** NHS participant characteristics (semi-Structured Interviews)

**Characteristic**	**Number of participants (*n*** **= 23)**
African region of birth	
East Africa	9
West Africa	11
Southern Africa	0
Middle Africa	3
Duration of residence in UK (years)	
<1	1
1–4	4
5–10	12
>10	6
Immigration status*	
Secure	15
Insecure	8
Diagnosis of HIV	
Prior to current pregnancy	20
During current pregnancy	3

*Secure immigration status is defined as being a UK citizen, a recognised refugee or having exceptional or indefinite leave to remain. Anyone not in these categories is defined as having insecure immigration status.

The first author conducted semi-structured interviews with 4 healthcare providers involved in the care of pregnant women living with HIV. They were recruited from the collaborating NHS sites and were invited to participate by the first author. They included two consultants in HIV medicine, one HIV specialist midwife and one specialist nurse in genitourinary medicine. Interviews were also conducted with two members of staff from voluntary sector organisations with experience of supporting African women living with HIV. These participants were identified through the first author’s knowledge of local voluntary sector organisations and were invited to participate by her. The purpose of interviewing health care professionals and voluntary sector workers was to elicit their experience of supporting this group of women and to identify what they saw as barriers to accessing care.

The first author also attended multidisciplinary meetings of healthcare professionals and observed some daily work at the antenatal clinics. These observations were recorded as field notes and were used to deepen and contextualise understanding of the interview data.

#### Ethnography

Ethnography consists of a combination of participant observation (observing activity whilst engaging directly with the world being studied), informal conversation, and formal interviews, within a social group. It can contribute to a rich and multidimensional understanding of social phenomena in groups [35]. Two field sites were selected for this study.

The first site was Body & Soul; a London-based charity that has been supporting children and families affected by HIV since 1996. A substantial number of African woman living with HIV who have experience of pregnancy attend Body & Soul. The first author worked at Body & Soul as a volunteer worker between April 2010 and December 2011, completing nearly 90 hours of participant observation. This fieldwork allowed the research team to explore the lived experience of people living with HIV, including some who were pregnant, in a non-clinical setting.

The second site was a Pentecostal church in London which has a largely diasporic Nigerian clergy and congregation. This choice of field site was guided by quantitative findings and initial interview data and this particular church was selected as it had been mentioned by a number of participants. The first author attended church services between July and September 2011, conducting nearly 40 hours of participant observation. This was complemented by watching broadcast footage of services, and conducting in-depth interviews with members of the congregation and people from the local community who are familiar with the church. The focus of this fieldwork was the role of Pentecostal faith in migrant Africans’ lives and how this particular church influenced attitudes towards parenthood, health and wellbeing.

### Ethics

The NSHPC has London Multi-Centre Research Ethics Committee approval (MREC/04/2/009). SOPHID does not require ethical approval as it fulfils a surveillance purpose. The HPA is registered under the Data Protection Act 1998 (registration number Z7749250) to handle data for diagnostic, public health and other purposes. The HPA is also registered under Section 251 of the Health and Social Care Act 2001 and has approval from the Patient Information Advisory Group (PIAG) to handle data for purposes that include surveillance and the control of disease, even where specific patient consent has not been given. Section 251 is renewed annually [36].

The qualitative phase of the study has ethical approval from City University Research Ethics Committee for qualitative research conducted outside NHS sites (ref PhD/09-10/10). It also has approval from the West London Research Ethics Committee for the overall qualitative phase, including on behalf of the NHS sites (ref 10/10707/49).

### Data analysis

This study collected both quantitative and qualitative data. Each dataset has been kept analytically distinct and has been analysed using appropriate techniques. We have moved between the datasets at the analysis stage to use findings from each analysis to generate hypotheses to be explored in the other datasets [37]. Linking will also occur at the interpretation stage, when results from the quantitative and qualitative analyses will be compared, contrasted and combined [38].

#### Quantitative analysis

Data were analysed using Stata 11.2 (Stata Corporation, College Station, Texas, USA). Data were summarized and examined for improbable values which were then checked against written records and amended accordingly or coded as missing. The dataset was checked for duplicate entries. Records with missing exposure or outcome data were excluded from analysis for the exposure and outcome of interest. Records with missing data on confounding variables were also dropped from final multivariable models. For analyses of trends we used the Bonferroni correction: this accounts for multiple comparisons within a group by adjusting the statistical significance level used for each test, minimising the chance of spurious positive results. For a given outcome, a Chi-square test was performed to compare pregnancies across different ethnicity, regional and migration groups. These groups were then compared for each outcome using univariable and multivariable logistic regression models to estimate odds ratios and adjusted odds ratios, with 95% confidence intervals. A priori confounders were included in final multivariable models. Other variables were included if their inclusion improved model fit. This was assessed using likelihood ratio tests, with a significance level of *p* < 0.05. We used robust standard errors where appropriate to account for potential clustering at a maternal level in sequential pregnancies for some outcomes.

#### Qualitative analysis

A professional transcription company transcribed all interviews, with quality checks undertaken by the first author. All interview transcripts and notes made during the fieldwork at Body & Soul were entered into NVivo 9. This qualitative data analysis software facilitates the classifying, sorting and linking of qualitative data. We are undertaking a thematic analysis of interview data, using the constant comparative method usually associated with grounded theory [39]. This is an inductive process where each transcript is read several times and sections of the text coded within the database. Coded text are then compared and linked across all the interviews if they capture a similar theme, leading to the development of broader key categories. We will pay particular attention to both the context of coded text, and also to data which does not appear to fit into the emerging thematic framework, in order to deepen our understanding. Some a priori codes will be developed from the quantitative phase, allowing us to interrogate the qualitative data to provide insight into our quantitative findings.

The first author made extensive written field notes during ethnographic fieldwork conducted at both Body & Soul and the Pentecostal church. Ethnographic research at the church also included in-depth interviews with members of the congregation and a local Pentecostal pastor (from a different local church). We also analysed church publications and recordings of television broadcasts of church services. All ethnographic data will be hand coded, using a manual index system to organise the data. We will begin with open coding, a process where codes are identified from the data without restriction, developing broader thematic categories using the constant comparative method.

The coding of transcripts and ethnographic data will be discussed with another member of the research team to improve rigour and reliability of the analysis.

### Advisory group

An advisory group was set up to provide guidance and support throughout the study. The group comprised: lay members; clinicians from the collaborating NHS centres; academics with an interest in HIV in African migrant groups; and representatives from Body & Soul and Positively UK.

## Discussion

This ongoing mixed-methods study has used epidemiological and anthropological methods to explore outcomes and experiences of pregnancy in African women living with HIV in the UK. Its particular strength is the innovative combination of quantitative and qualitative approaches, which will enable a richer understanding of this complex and multi-faceted area. Although mixed methods are increasingly used in health services research, methods such as secondary analysis of surveillance datasets and ethnography are rarely used in the context of mixed methods research. In a recent review O’Cathain et al. [40] found that the quantitative component in mixed methods health services research largely comprised primary data collection through surveys, other observation studies or intervention studies. Furthermore, semi-structured interviews were the qualitative method of choice in 80% of studies, with participant observation described in less than 1%.

The surveillance dataset used in this study was not designed for our research question and there were therefore no data on key variables such as socioeconomic and immigration status. Furthermore women interviewed in the qualitative phase may not have been included in the surveillance dataset. However, given that almost all pregnant women living with diagnosed HIV are reported to the NSHPC, it is unlikely that findings from the quantitative phase would not apply to women recruited in the qualitative phase and vice versa. The advantage of using surveillance data is the statistical power gained from such large numbers, generalisability, and the efficiency in time. The ethnographic component, although limited in duration as a result of the mixed-methods design, has resulted in a rich understanding of women’s lives [35]. The findings may also allow us to inform future HIV surveillance data collection by identifying potential factors that may impact on pregnancy that are currently not collected. We feel that the methodology used in this study could be applied to other settings where complex public health questions arise.

We anticipate that the data obtained from this study will inform the provision of care to pregnant women living with HIV and the development of services that prioritise and address their needs, leading to improvements in maternal and child health.

## Abbreviations

HIV, Human immunodeficiency virus; ART, Antiretroviral therapy; MSM, Men who have sex with men; MTCT, Mother-to-child transmission.

## Competing interests

The authors declare that they have no competing interests.

## Authors’ contributions

ST conceived the study. ST designed the study with input from JE, AP and PT. AB and PT supervised the linkage of surveillance datasets. ST was responsible for qualitative data collection, and the data management and analyses of both quantitative and qualitative data. JE, AB, AP and PT provided supervision and guidance on analyses and conduct of the research. ST drafted the manuscript with input from JE, AB and PT. All authors read, revised and approved the final manuscript.

## Authors’ information

S Tariq is currently funded by the UK Medical Research Council (MRC) (Award number: G0701648 ID 85538) administered by City University London. The NSHPC receives core funding from the Health Protection Agency, and is located in the Centre for Paediatric Epidemiology and Biostatistics, which benefits from the MRC in its capacity as the MRC Centre of Epidemiology for Child Health. The University College of London (UCL) Institute of Child Health receives a proportion of funding from the Department of Health’s National Institute for Health Research Biomedical Research Centres funding scheme. Any views expressed in this paper are those of the authors, and not necessarily those of the funders.

## Pre-publication history

The pre-publication history for this paper can be accessed here:

http://www.biomedcentral.com/1471-2458/12/596/prepub
